# Multiple Biogenic Amine Receptor Types Modulate Spider, *Cupiennius salei*, Mechanosensory Neurons

**DOI:** 10.3389/fphys.2018.00857

**Published:** 2018-07-09

**Authors:** Vaishnavi Sukumar, Hongxia Liu, Shannon Meisner, Andrew S. French, Päivi H. Torkkeli

**Affiliations:** Department of Physiology and Biophysics, Dalhousie University, Halifax, NS, Canada

**Keywords:** octopamine, tyramine, transcriptome, phylogeny, mechanosensory, arachnid

## Abstract

The biogenic amines octopamine (OA), tyramine (TA), dopamine (DA), serotonin (5-HT), and histamine (HA) affect diverse physiological and behavioral processes in invertebrates, but recent findings indicate that an additional adrenergic system exists in at least some invertebrates. Transcriptome analysis has made it possible to identify biogenic amine receptor genes in a wide variety of species whose genomes have not yet been sequenced. This approach provides new sequences for research into the evolutionary history of biogenic amine receptors and allows them to be studied in experimentally accessible animal models. The Central American Wandering spider, *Cupiennius salei*, is an experimental model for neurophysiological, developmental and behavioral research. We identified ten different biogenic amine receptors in *C. salei* transcriptomes. Phylogenetic analysis indicated that, in addition to the typical receptors for OA, TA, DA, and 5-HT in protostome invertebrates, spiders also have α1- and α2-adrenergic receptors, but lack TAR2 receptors and one invertebrate specific DA receptor type. *In situ* hybridization revealed four types of biogenic amine receptors expressed in *C. salei* mechanosensory neurons. We used intracellular electrophysiological experiments and pharmacological tools to determine how each receptor type contributes to modulation of these neurons. We show that arachnids have similar groups of biogenic amine receptors to other protostome invertebrates, but they lack two clades. We also clarify that arachnids and many other invertebrates have both α1- and α2-adrenergic, likely OA receptors. Our results indicate that in addition to an OAβ-receptor that regulates rapid and large changes in sensitivity via a G_s_-protein activating a cAMP mediated pathway, the *C. salei* mechanosensory neurons have a constitutively active TAR1 and/or α2-adrenergic receptor type that adjusts the baseline sensitivity to a level appropriate for the behavioral state of the animal by a G_q_-protein that mobilizes Ca^2+^.

## Introduction

The biogenic amines histamine (HA), dopamine (DA), and serotonin (5-HT) are common neurotransmitters in both vertebrates and invertebrates, but adrenergic signaling has been considered absent in protostome invertebrates, where monoamine octopamine (OA) and its precursor tyramine (TA) have replaced noradrenaline and adrenaline (Roeder, 2005; Lange, 2009; Ohta and Ozoe, 2014; Blenau et al., 2017a,b). Biogenic amines act mainly on G-protein coupled receptors (GPCRs) and arthropod receptors are classified based on similarities in their molecular structures and signaling pathways to their vertebrate counterparts (Evans and Maqueira, 2005; Verlinden et al., 2010; Ohta and Ozoe, 2014). Two arthropod receptor types have the highest affinity to OA: (1) α1-adrenergic-like OA receptors (OARα), coupled to G_q_ proteins that activate phospholipase-C (PLC), leading to mobilization of intracellular Ca^2+^ and, to a lesser extent, G_s_ proteins that activate adenylate cyclase and stimulate cAMP production. (2) β-adrenergic-like OA receptors (OARβ), acting only on G_s_ proteins to stimulate cAMP production (Balfanz et al., 2005; Evans and Maqueira, 2005; Verlinden et al., 2010; Ohta and Ozoe, 2014). Two other receptor types in many arthropods have their highest affinity to TA: (1) TAR1 receptors are structurally closest to vertebrate α2-adrenergic receptors, activated by both TA and OA (Lange, 2009). They signal by inhibiting cAMP production via G_i_ proteins and/or activate G_q_ proteins to elevate [Ca^2+^] (Evans and Maqueira, 2005). (2) TAR2 receptors are specific to TA and coupled to G_q_ proteins and/or G_s_ proteins (Cazzamali et al., 2005; Bayliss et al., 2013; Wu et al., 2015; Reim et al., 2017).

Recent research has characterized several invertebrate receptors that are phylogenetically closer to vertebrate α1- or α2-adrenergic receptors than any previously studied OA or TA receptors (Wu et al., 2014; Bauknecht and Jekely, 2017; Qi et al., 2017). When expressed in cell lines, some of these receptors respond to adrenaline and/or noradrenaline suggesting that the evolutionary history of adrenergic signaling may not have been as direct as previously thought (Bauknecht and Jekely, 2017).

Classification of other invertebrate G-protein coupled biogenic amine receptors is also based on their similarities with vertebrates: For dopamine there are DAR1 or DAR2 receptors, with the former group coupled to G_s_ proteins, and the latter group to G_i_ proteins (Blenau and Baumann, 2001). For serotonin, arthropods have two types of 5-HT_1_ (coupled to G_i_), two types of 5-HT_2_ (coupled to G_q_), and one 5-HT_7_ (coupled to G_s_) receptor (Thamm et al., 2010, 2013; Blenau et al., 2017b). So far, histamine activated GPCRs have not been identified in arthropods, but HA acts via Cl^-^ selective ion channels.

Transcriptome analysis has made it possible to discover biogenic amine receptor sequences in animal species whose genomes have not yet been sequenced, allowing re-evaluation of their evolutionary history in a larger range of species. Although many invertebrate biogenic amine receptors have been extensively investigated in expression systems, providing useful information about their signaling pathways and pharmacology, it has been more difficult to test these receptors in their native environments. Identification of specific receptor types from experimentally accessible animal models makes it possible to determine the physiological functions of these receptors in the cells and tissues where they are normally expressed.

Most research into invertebrate biogenic amine receptors has used insect models because they provide targets for insecticide development. However, beneficial insects and other arthropods, including natural predators of insects such as spiders, have homologous receptors. The Central American wandering spider, *Cupiennius salei*, has served as a model in neurophysiological, developmental, and behavioral research. Its central nervous system has OA, HA, and 5-HT immunoreactive neurons (Schmid and Duncker, 1993; Seyfarth et al., 1993; Barth, 2002) and its mechanosensory neurons were also immunoreactive to HA (Fabian and Seyfarth, 1997). These neurons receive extensive efferent innervation, and some efferent fibers were immunoreactive to OA (Widmer et al., 2005). In electrophysiological experiments, the mechanosensory neurons respond to OA with a surge in sensitivity and there is evidence for involvement of both cAMP and Ca^2+^-mediated pathways (Widmer et al., 2005; Torkkeli et al., 2011). Here, we identified ten sequences from *C. salei* transcriptomes that code putative biogenic amine receptors and performed phylogenetic analysis to determine their evolutionary relationships. We then created RNA probes for *in situ* hybridization to locate genes expressed in the spider mechanosensory neurons. To discover how activation or inhibition of each of these receptors modulate mechanosensory neurons, we performed intracellular recordings while testing the effects of several agonists and antagonists on the neurons of the lyriform VS-3 slit sensilla that detects strain and vibration in the spider leg patella (Barth, 2002; French et al., 2002).

## Materials and Methods

### Experimental Animals and Dissection

A breeding colony of the Central American wandering spiders, *C. salei* (Keys.) were maintained at 22°C ± 2°C under a 13 light and 11 h dark cycle. Autotomized legs from male and female adult spiders were used for experiments following a protocol approved by the Dalhousie University Committee on Laboratory Animals. Legs were pinned down on a Sylgard (Dow Corning, Auburn, MI, United States)-coated dish. For *in situ* hybridization and electrophysiology, the anterior patella that contains the lyriform slit sensilla VS-3 (Barth, 2002) and several tactile hair sensilla, was dissected from the leg. The muscles were removed to reveal the leg nerves and sensory neurons that are attached to a hypodermis membrane directly beneath the cuticle.

### Identification of Biogenic Amine Receptors From Spider Transcriptomes

Detailed description of the *C. salei* hypodermis and brain transcriptome preparation, sequencing, and assembly has been published previously (French, 2012; Torkkeli et al., 2015). Putative biogenic amine receptor sequences were identified using the transcriptome walking algorithm (French, 2012). We could not complete one sequence (Csα2-adrenergic receptor) from the transcriptome data. The full sequence was obtained by rapid amplification cDNA ends (RACE) using the methods described by Scotto-Lavino et al. (2006). The gene specific primer for the first round PCR was TCTTCGTGTACTGTCGCATC and the primer for second round PCR was AGTCAACAACCACGACCAC. The sequencing was done by McGill University and Génome Québec Innovation Centre (Montréal, QC, Canada).

We estimated the relative abundance of each sequence by counting matching Illumina reads as described earlier (French, 2012; Torkkeli et al., 2015). Data analysis and programming were performed using the C++ language with Microsoft Visual Studio and desktop computers.

### Phylogenetic Analysis

We searched homologous sequences for each of the ten *C. salei* putative biogenic amine receptors using protein Delta-Blast. Sequence alignment was performed using multiple sequence alignment software MAFFT Version 7^1^ with default parameters using G-Ins-I strategy with threshold E = 1*e*^-10^ (Katoh and Standley, 2013). The final phylogenetic tree was created with MEGA 7 software using the Maximum likelihood method (Kumar et al., 2016). The Gamma distributed Le-Gascuel model (Le and Gascuel, 2008) that allowed some sites to be evolutionarily invariable (LG + G + I) was used. The final analysis involved 149 amino acid sequences and we used 1,000 bootstraps with strong subtree pruning and partial deletion with otherwise default settings. *C. salei* sequence accession numbers are shown in **Table 1** and sequences from other species are listed in the Supplementary Table 1. The final image was created in Adobe illustrator CC software (Adobe Systems Inc., San Jose, CA, United States).

**Table 1:** *Cupiennius salei* biogenic amine receptor nomenclature and primer sequences used for RNA probes for *in situ* hybridization.

Protein	Accession #	Probe size (bp)	Primers
Csα1-Adrenergic	GBFC01000002.1	621	AS: GGATTCACCTCTAACCTTCGTAGCAC *TAATACGACTCACTATAGGGGTACGTTCCGTTACGAGCAGCAG* S: *TAATACGACTCACTATAGGGGGATTCACCTCTAACCTTCGTAGCAC* GTACGTTCCGTTACGAGCAGCAG
Csα2-Adrenergic	MH480604	554	AS: CAGTTCGCTGGGAGAGATATGAAGT *TAATACGACTCACTATAGGGCATTGCTGAAGCTCGTGCATG* S: *TAATACGACTCACTATAGGGCAGTTCGCTGGGAGAGATATGAAGT* CATTGCTGAAGCTCGTGCATG
CsOARα	GBFC01000014.1	532	AS: CATACTTGTACCTCGTCGTATCCC *TAATACGACTCACTATAGGGGAATGAGCGATGTCACACCATC* S: *TAATACGACTCACTATAGGGCATACTTGTACCTCGTCGTATCCC* GAATGAGCGATGTCACACCATC
CsOARβA	GAKT01000098.1	597	AS: CACCACTGGCAGAAACCGCATT *TAATACGACTCACTATAGGGCCATCTCCCGTACAAAGTGTTCGTC* S: *TAATACGACTCACTATAGGGCACCACTGGCAGAAACCGCATT* CCATCTCCCGTACAAAGTGTTCGTC
CsOARβB	GBFC01000017.1	582	AS: AAGGCAGGGATTATTCCAAACG *TAATACGACTCACTATAGGGCAGTCCTCAACGATAATACGACAC* S: *TAATACGACTCACTATAGGGAAGGCAGGGATTATTCCAAACG* CAGTCCTCAACGATAATACGACAC
CsTAR1	GBFC01000018.1	605	AS: GTATTGGGCAATACACGATCCAAT *TAATACGACTCACTATAGGGGTCTAACAGTGGCTGTTAGCCTTGG* S: *TAATACGACTCACTATAGGGGTATTGGGCAATACACGATCCAAT* GTCTAACAGTGGCTGTTAGCCTTGG
CsDAR1	GBFC01000016.1	682	AS: CTCGTGTCCTTTCTTCCTATCAGTC *TAATACGACTCACTATAGGGATTTCCCAGACCACTCTCCTTGC* S: *TAATACGACTCACTATAGGGCTCGTGTCCTTTCTTCCTATCAGTC* ATTTCCCAGACCACTCTCCTTGC
CsDAR2A	MG981030	718	AS: CATTCTCCGGGCGCAGATAGCGA *TAATACGACTCACTATAGGGTGGCTGCGAGCACACGAGCATT* S: *TAATACGACTCACTATAGGGCATTCTCCGGGCGCAGATAGCGA* TGGCTGCGAGCACACGAGCATT
CsDAR2B	MG981031	546	AS: AGGATGACGAATCGCCACCT *TAATACGACTCACTATAGGGGCTGGAAGAGGAGTTTCTTGAAGG* S: *TAATACGACTCACTATAGGGAGGATGACGAATCGCCACCT* GCTGGAAGAGGAGTTTCTTGAAGG
Cs5-HTR_1_	GBFC01000015.1	718	AS: CACAGCGATACTTATAGTTTGGACAG *TAATACGACTCACTATAGGGGGTGTAAATAATGGGATTGAGCG* S: *TAATACGACTCACTATAGGGCACAGCGATACTTATAGTTTGGACAG* GGTGTAAATAATGGGATTGAGCG

*OARα, octopamine α receptor; OARβ, octopamine β receptor; TAR1, tyramine receptor Type 1; DAR1, dopamine receptor Type 1; DAR2, Dopamine receptor Type 2; 5-HTR_*1*_, serotonin receptor type 1; AS, antisense; S, sense. The T7 promoter sequences are shown in italics.*

### RNA Probe Construction and *in Situ* Hybridization

The primers (Integrated DNA Technologies, Coralville, IA, United States) used to create *in situ* hybridization probes are listed in **Table 1**. Digoxigenin-labeled sense and antisense RNA probes were transcribed *in vitro* from previously obtained cloning plasmids of spider hypodermis cDNA with T7 RNA polymerase (Roche Diagnostics, Laval, QC, Canada; Liu et al., 2017) following protocols recommended by the manufacturer. Probe quality was confirmed by agarose gel electrophoresis. The probes were then stored at -80°C until use. The whole-mount *in situ* hybridization protocol was performed as previously described in detail (Liu et al., 2017). The complete experimental protocols were repeated at least twice for each probe, every time using eight to 10 legs. Finally, the hypodermis was carefully detached from the cuticle and mounted on a microscope slide in 70% glycerol. Samples were inspected and photographed with a compound microscope (Olympus BH-2, Richmond Hill, ON, Canada) equipped with a digital camera (MU500; AmScope, Irvine, CA, United States), and the images were processed using Adobe Photoshop CC software (Adobe Systems).

### Intracellular Electrophysiology

Electrophysiological experiments from the VS-3 neurons in the hypodermis preparation were performed as described before (Sekizawa et al., 1999). Bath solution contained normal spider saline (223 mM NaCl, 6.8 mM KCl, 8 mM CaCl_2_, 5.1 mM MgCl_2_, and 10 mM HEPES, pH 7.8) and the flow rate of the superfusion was maintained at about 1 mL/min. All chemicals were purchased from Sigma (Oakville, ON, Canada) if not otherwise indicated. VS-3 organs were observed under a compound microscope with a 10× objective (Axioskop 2FS Plus; Zeiss, Oberkochen, Germany). The microscope was mounted on a gas-driven vibration isolation table inside a Faraday cage (Technical Manufacturing, Peabody, MA, United States). Sharp borosilicate glass microelectrodes (outside diameter, 1 mm; inside diameter, 0.5 mm; World Precision Instruments, Sarasota, FL, United States), pulled with a P-2000 horizontal laser puller (Sutter Instruments, Novato, CA, United States) were filled with 3 M KCl, and had resistances of 40–80 MΩ in solution. Microelectrodes were placed into the neurons using PatchStar micromanipulators (Scientifica, Uckfield, United Kingdom). Recordings were performed in discontinuous current-clamp mode, with a SEC-10L amplifier (NPI Electronic, Tamm, Germany), as described previously in detail (Sekizawa et al., 1999; Pfeiffer et al., 2009).

Agonists were applied manually with a syringe via separate plastic tubing into the superfusion solution. The following agonists were tested: octopamine (OA) hydrochloride, tyramine (TA) hydrochloride, dopamine (DA) hydrochloride, histamine (HA) dihydrochloride, and 5-hydroxytryptamine (5-HT). The total amount of agonists injected was 100–200 μL. Biogenic amine receptor antagonists were added to the superfusion solution for a minimum of 30 min and up to 2 h, and the neuron’s responses to agonists were tested every 15 min while the antagonist was present. The following antagonists were tested: Yohimbine hydrochloride, chlorpromazine hydrochloride, mianserin hydrochloride, metoclopramide hydrochloride, and phentolamine hydrochloride. Concentrated solutions of each agonist and antagonist were stored frozen in double distilled water, except phentolamine, which was dissolved initially in 100% ethanol.

### Statistical Analysis

Experiments with each agonist and antagonist were repeated with each concentration in a minimum of five and up to 33 neurons. Before statistical analysis, we used the D’Agostino and Pearson test to assess whether the data were normally distributed. At least five independent experiments were performed for each agonist and antagonist. When comparing more than two groups with normal distribution, one-way ANOVA with Tukey HSD Test was performed. Non-parametric Kruskal–Wallis multiple-comparison test was used on the data distributions that did not pass the normality test with *p* = 0.05. Comparison of two conditions was done by paired or unpaired *t*-tests for normally distributed pairs or non-parametric Mann–Whitney test for data that did not pass the normality test. All tests are stated in Figure legends. Results from directional tests are reported. The tests were performed using VassarStats software^2^. Statistical significances in figures are indicated as asterisks: ^∗^*p* ≤ 0.05, ^∗∗^*p* ≤ 0.01, and ^∗∗∗^*p* ≤ 0.001.

## Results

### Biogenic Amine Receptors in *C. salei* Central Nervous System (CNS) and Hypodermis Transcriptomes

The deduced proteins from ten mRNA sequences in *C. salei* CNS and hypodermis transcriptomes had conserved features of biogenic amine receptors. InterPro scan recognized all ten proteins as 7-transmembrane helix GPCRs. Nine of these were complete sequences but one could not be completely assembled from the transcriptome. We used the Rapid amplification of cDNA ends (RACE) to obtain the full-length sequence for this transcript. Based on conserved motifs and phylogenetic analysis (below) the sequences were named as indicated in **Table 1**.

**FIGURE 1:**
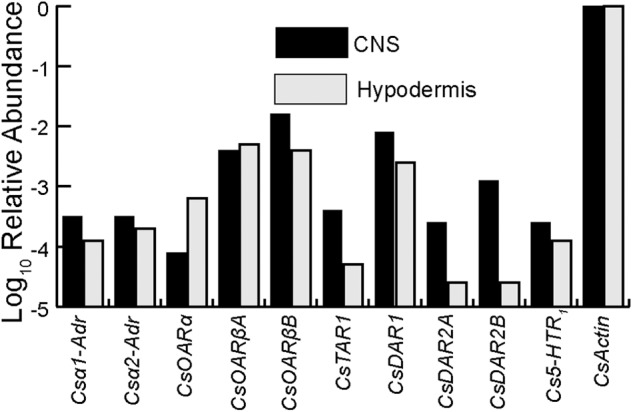
Relative abundances of biogenic amine receptors in the *C. salei* CNS and hypodermis transcriptomes. The data were obtained by counting total reads in the transcriptome libraries with at least 90 consecutive identical nucleotides to the reading frame of each gene, then normalizing by reading frame length. Abundances in CNS are indicated in black while the hypodermis is indicated in light gray. The scale is logarithmic. Accession numbers and sequence nomenclature are shown in **Table 1**.

The relative abundance of transcribed mRNA for each putative biogenic amine receptor sequence was estimated by searching the transcriptome data for matches to the main open reading frame (mORF) as explained previously in detail (French, 2012; Torkkeli et al., 2015). The total counts were normalized by mORF length, expressed as abundance relative to the putative actin coding gene. The relative abundances of each *C. salei* biogenic amine receptor in the CNS and hypodermis transcriptomes are shown in **Figure 1**. All ten sequences were found in both tissues, and generally in similar amounts, but the relative abundances of some (*CsTAR1, CsDAR2A*, and *CsDAR2B*) were higher in the CNS than in the hypodermis while other sequences were more abundant in the hypodermis (*CsOARβA* and *CsOARα*). Both the CNS and hypodermis tissues that were used to create the transcriptomes contained neurons and glial cells, but some muscle tissue was also attached to these structures and it is likely that some of the biogenic amine receptor sequences originated from muscle tissue.

### Molecular Phylogenetic Analysis

**FIGURE 2:**
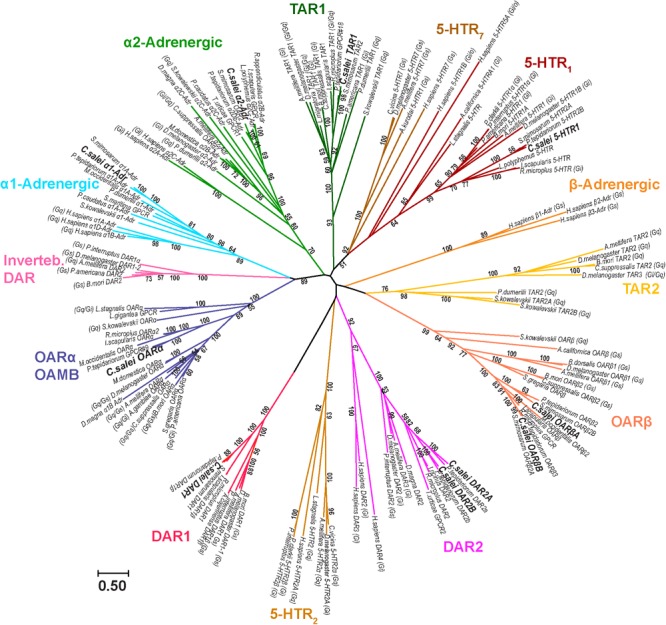
Molecular phylogenetic analysis of putative biogenic amine receptors. Evolutionary history was inferred by the Maximum Likelihood method based on the Le-Gascuel (Le and Gascuel, 2008) model. The unrooted radiation tree is drawn to scale, with branch lengths measured in numbers of substitutions per site. The analysis involved 149 MAFFT aligned amino acid sequences and was run with 1,000 bootstraps. Numbers indicate bootstrap values. There were 1,385 positions in the final dataset. Evolutionary analyses were conducted in MEGA7. Insects: *Anopheles gambiae, Apis mellifera, Bactrocera dorsalis, Bombyx mori, Calliphora vicina, Chilo suppressalis, Drosophila melanogaster, Musca domestica, Locusta migratoria, Periplaneta americana, Schistocerca gregaria*. Chilopoda: *Strigamia maritima*. Arachnids, ticks: *Amblyomma cajennense, Ixodes ricinus, Ixodes scapularis, Rhipicephalus appendiculatus, Rhipicephalus microplus.* Mites: *Metaseiulus occidentalis, Tetranychus urticae.* Spiders: *Parasteatoda tepidariorum, Stegodyphus mimosarum*. Crustacea: *Daphnia magna, Panulirus interruptus, Procambarus clarkii.* Merostomata: *Limulus polyphemus.* Annelida: *Platynereis dumerilii*. Priapulida: *Priapulus caudatus*. Mollusca: *Aplysia californica, Aplysia kurodai, Lottia gigantea, Lymnaea stagnalis.* Hemichordata: *Saccoglossus kowalevskii*. Chordata: *Homo sapiens.* Accession numbers and nomenclature for *C. salei* sequences are in **Table 1** and for sequences of other species in the Supplementary Table 1. The G-protein coupled pathways (G_i_, G_s_, G_o_, G_q_) are indicated for sequences where such information was available.

The evolutionary relationships of *C. salei* biogenic amine receptors to homologous sequences in other species was investigated by molecular phylogenetic analysis based on the Maximum likelihood algorithm. Each *C. salei* biogenic amine receptor sequence clustered with high bootstrap values into orthologous clades in various invertebrate phyla. The closest homologs were generally found in arachnids. Some *C. salei* sequences are also orthologous to mammalian biogenic amine receptors. The radiation tree (**Figure 2**) includes representative examples of arthropod and human sequences. For those receptors where the G-protein coupled pathways have been confirmed in heterologous expression systems, these pathways are indicated in **Figure 2**.

One of the *C. salei* OA receptors is in the α-adrenergic-like OARα clade that includes well characterized insect OAMB receptors (OA receptor in mushroom bodies) that are usually coupled to G_q_ and/or G_s_ proteins (Han et al., 1998; Balfanz et al., 2005). However, *C. salei* and other invertebrates also have receptors in the same clades with human α1- and α2-adrenergic receptors (coupled to G_q_ and G_i_, respectively). One *C. salei* sequence is in the tyramine 1 (TAR1) clade that has several invertebrate receptors that, in expression systems, are activated by both TA and OA and are coupled to G_i_ and/or G_q_ proteins (Verlinden et al., 2010; Ohta and Ozoe, 2014). We did not find any *C. salei* sequences in the TAR2 clade and GenBank did not have arachnid receptors in this clade either, suggesting that these stimulatory tyramine receptors are absent in arachnids. Two of the *C. salei* sequences were in the OARβ clade that has many invertebrate receptors that are coupled to G_s_ proteins and have been shown to stimulate cAMP production (Verlinden et al., 2010; Ohta and Ozoe, 2014). Our analysis shows that the invertebrate β-adrenergic receptors are phylogenetically distant from the human β-adrenergic receptors. Two of the *C. salei* receptors are in the DAR2 clade that has many invertebrate receptors and is relatively close to human DAR2 group (DAR2, 3, and 4), all of which act on inhibitory G_i_ proteins. *C. salei* has one receptor that is orthologous to the invertebrate DAR1 receptors that are coupled to G_s_ proteins. In our analysis, the invertebrate DAR1 sequences were not closely related to the vertebrate DAR1 group that is not included into the final tree. Some invertebrate dopamine receptors clustered to a clade close to the α1-adrenergic receptors, but this clade has no arachnid representatives. One *C. salei* receptor was in a serotonin clade with other arachnids, close to the insect 5-HT_1_ sequences that have been shown to act by inhibiting cAMP production via G_i_ protein.

### Expression of Biogenic Amine Receptors in Mechanosensory VS-3 Neurons

**FIGURE 3:**
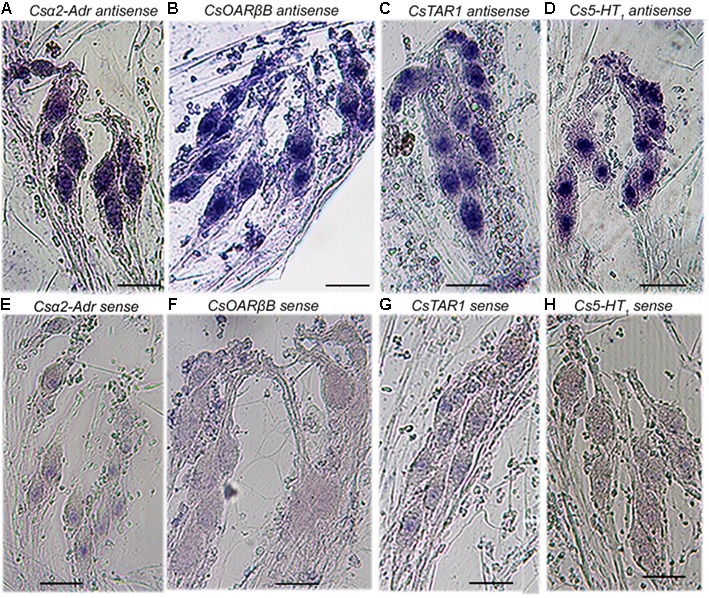
Expression of four putative biogenic amine receptors in the mechanosensory neurons of VS-3 slit sensilla. Whole-mount *in situ* hybridization of the patellar hypodermis using digoxigenin labeled antisense and sense (control) probes. Antisense RNA probes for putative *Csα2-Adrenergic* **(A)**, *CsOARβB* **(B)**, *CsTAR1* **(C)**, and *Cs5-HT_1_* **(D)**, receptors produced strong signals in VS-3 neurons, while the sense probes for the same genes did not **(E–H)**. Scale bars 50 μm in all images.

Transcripts for all ten biogenic amine receptors were found in both *C. salei* brain and peripheral nervous system transcriptomes (**Figure 1**). To learn which receptors were expressed in mechanosensory neurons, we tested specific mRNA probes for each receptor (**Table 1**) using *in situ* hybridization on the hypodermis of the leg patella. The hypodermis is located beneath the cuticle and consists of epithelial, glial and pigment cells and the leg nerves with numerous sensory, motor, and other efferent branches (Liu et al., 2017). The part of the patella used here contains several mechanosensory organs including trichoid hair sensilla and the mechanosensory lyriform slit sensilla VS-3 that has 7–8 pairs of sensory neurons. Each mechanosensory neuron is enwrapped by glial cells (Liu et al., 2017). Four antisense probes for biogenic amine receptors produced strong signals in the patellar mechanosensory neurons. **Figure 3** shows labeling in the neurons of the VS-3 organ with antisense probes for the *C. salei* α2-adrenergic (*Csα2-Adr*), serotonin (*Cs5-HT_*1*_*), tyramine (*CsTAR1*), and one of the two octopamine-β receptors (*CsOARβ2*). Similar labeling was present in all other mechanosensilla in the patellar hypodermis, but the glial cells, epithelial, and pigment cells were not labeled. Sense probes for the same genes did not produce labeling in the cell cytoplasm, but in some cases, there was slight nuclear labeling. Antisense probes for the other six probes (**Table 1**) did not produce specific labeling.

### Pharmacology of Biogenic Amine Receptors in the VS-3 Neurons

Our finding of expression of at least four different biogenic amine receptor types in the spider mechanosensory neurons, suggests that several biogenic amines may modulate these neurons and physiological effects of each of them may vary depending on which receptor types they activate. Homologous receptors from other arthropod species have been expressed in cell lines and the G-proteins that they activate have been identified. Therefore, the expected effect of activation of OARβ is to activate G_s_ proteins and stimulate the neurons (Maqueira et al., 2005; Balfanz et al., 2014). Most TAR1, α2-adrenergic, and 5-HT_1_ receptors are coupled to G_i_ proteins and inhibit cAMP production (Robb et al., 1994; Chen et al., 2004; Qi et al., 2017). However, some of these receptors are also coupled to G_q_ proteins that stimulate IP_3_ synthesis and mobilize calcium (Robb et al., 1994; Poels et al., 2001; Gross et al., 2015). Therefore, biogenic amines could have either excitatory or inhibitory effects on the spider mechanosensory neurons. Using intracellular recordings from the mechanosensory VS-3 neurons, we tested multiple agonists and antagonists to determine how they modulate the sensitivity of these neurons when they are stimulated to fire action potential using pseudorandom noise stimulation.

### Functional Effects of Biogenic Amines on VS-3 Neurons

**FIGURE 4:**
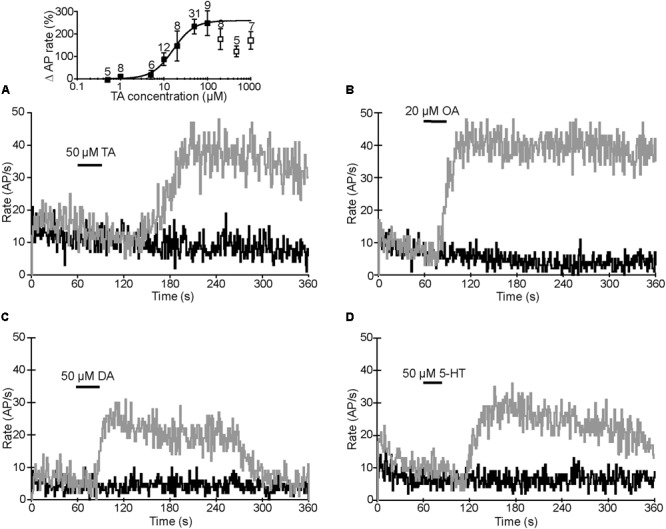
Agonist effects on action potential (AP) rate. Black trace in each image is the AP rate in control recording when the preparation was superfused in normal spider saline. Gray traces are the AP rates recorded from the same neurons when TA **(A)**, OA **(B)**, DA **(C)**, or 5-HT **(D)** were applied at the times indicated. APs were elicited by pseudorandom white noise electrical stimulation and converted to AP rates. **Inset in A:** dose–response relationship for TA. Percentage changes in spike rates are plotted against TA concentrations. The data was fitted with the logistic Hill equation Y =Y_max_[C]^n^/Y_max_[C]^n^([C]^n^ [EC_50_]^n^)([C]^n^ [EC_50_]^n^), where Y is response, Y_max_ is maximal response, [C] is agonist concentration, the half-maximal effective concentration [EC_50_] is 16.3 μM, and the Hill coefficient (n) is 1.8. The points used for the fit are shown as black squares. At concentrations above 100 μM, the TA effect was below the peak value (white squares) and could not be fitted. Results are shown as means ± SE with the number of experiments indicated above each symbol.

Initially, the preparations were superfused in spider saline and the VS-3 neurons were stimulated with pseudorandom white noise. We used noise stimulation because it contains a similarly wide bandwidth of frequency components to those that these neurons receive in nature (Pfeiffer et al., 2009). To quantify changes in spike rate, the original recordings were converted to impulses per second using 1-s wide bins as explained earlier in detail (Widmer et al., 2005; Pfeiffer et al., 2009). Depending on the stimulus, the neurons either fired steadily during the complete recording period or the spike rate was initially higher and then adapted to a plateau (**Figure 4**). For recordings with agonists, the drug application was made 60 s after the start of the experiment, as indicated in **Figure 4**. Several different concentrations of each agonist were tested, and the examples shown in **Figure 4** are at the saturating concentrations for each agonist that increased the spike rate: 50 μM TA, 20 μM OA, 50 μM DA, and 50 μM 5-HT. The percentage changes in firing rate for each agonist are shown in **Table 2**. Each agonist also produced a small depolarizing synaptic potential and their amplitudes are also listed in **Table 2**. Statistical analyses did not detect differences between the agonists for either effect. We also tested histamine, but did not detect any changes in VS-3 neuron firing rate or membrane potential with concentrations of up to 400 μM. We did not detect inhibitory effects on firing rate with any of the agonists tested. The speed of the agonist effect varied in different experiments, probably due to slight differences in the distance of the tubing to the neuron and/or the flow rate. This variation was similar for each agonist.

**Table 2:** Agonists effects on AP rate and membrane potential.

Agonist	AP rate change (%)	Synaptic potential (mV)
Tyramine (TA)	234 ± 188 (31)	5.0 ± 2.7 (31)
Octopamine (OA)	286 ± 243 (37)	4.8 ± 3.1 (36)
Dopamine (DA)	203 ± 154 (9)	3.7 ± 2.2 (9)
5-HT	332 ± 381 (7)	2.6 ± 2.0 (7)
Statistics	*H* = 0.78,	*F* = 1.88,
	*p* = 0.8542	*p* = 0.139727

*Statistical analysis for AP rate Kruskal–Wallis test, for Synaptic potential one-way ANOVA.*

We did not perform further tests with 5-HT or DA but concentrated on potential differences in the TA and OA responses. We determined the dose–response relationship for TA by measuring peak AP rates at different TA concentrations (inset in **Figure 4A**). The maximal effect was reached at 50–100 μM, but higher TA concentrations produced a smaller effect. Therefore, we only used the data between 100 nM and 100 μM for fitting with the logistic Hill equation. The Hill coefficient was 1.8 and half maximal concentration (EC_50_) 16.3 μM. The dose–response relationship for OA has been reported earlier (EC_50_ = 1.39 μM and Hill coefficient = 0.96) (Widmer et al., 2005) and the maximal effect was reached at 20 μM, indicating that OA is a more effective agonist than TA. Similar to the TA effect shown here, the OA effect has also been shown to be smaller at high concentrations (Torkkeli et al., 2011).

### Biogenic Amine Antagonist Effects on OA and TA Responses

**FIGURE 5:**
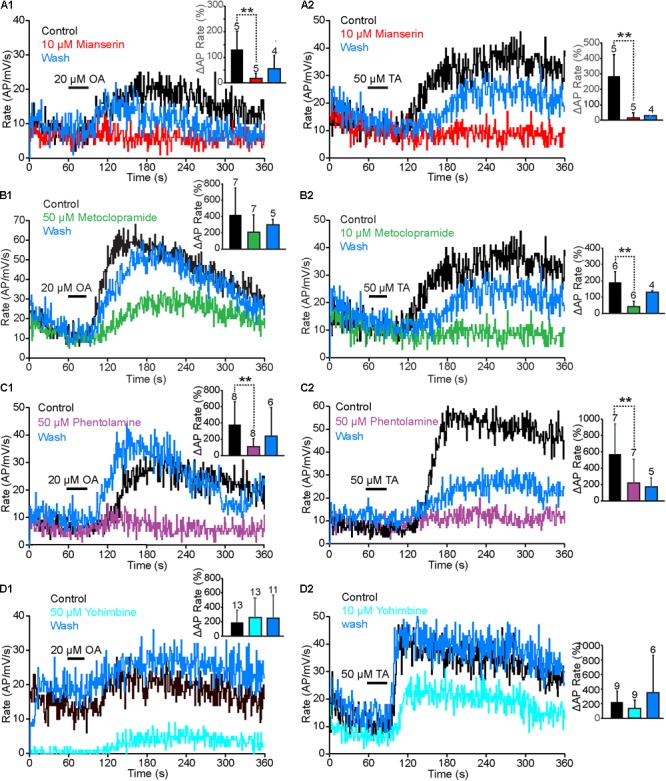
Antagonist effects on OA and TA responses. **(A1,A2)** The rise in OA or TA induced AP rate was smaller when mianserin was added into the superfusion than under control conditions. This effect was reversed partially after wash. **(A1,A2)** OA and TA induced changes in spike rate in mianserin treated neurons were statistically significantly smaller than under control conditions. (Paired *t*-tests. OA: *p* = 0.005495, *t* = 4.48, *df* = 4. TA: *p* = 0.0035905, *t* = 5.06, *df* = 4). (**B1,B2)** Increases of AP rates by OA and TA were larger under control conditions, than with metoclopramide in the superfusion. These effects recovered almost completely after wash. **(B1,B2)** The OA induced changes to AP rate by metoclopramide were not statistically significant (Paired *t*-test *p* = 0.0959795, *t* = 1.47, *df* = 6), but the TA induced change was statistically significant (Paired *t*-test, *p* = 0.003895, *t* = 4.29, *df* = 5). (**C1,C2)** Phentolamine strongly inhibited the VS-3 neurons response to OA and TA and this effect was at last partially reversible. **(C1,C2)** Phentolamine effects were statistically significant. (OA: paired *t*-test *t* = 4.03, *df* = 7, *p* = 0.002498. TA: Wilcoxon rank-sum test *W* = 28, *n*_s/r_ = 7, *p* = 0.01). **(D1)** Fifty micromolar yohimbine in the superfusion clearly reduced the neuron’s response to pseudorandom noise stimulus, but it still responded to OA with an increase in AP rate. After wash, the neuron responded to the same stimulus with higher spike rate than control and the response was amplified by OA application. **(D2)** Ten micromolar yohimbine also reduced the baseline spike rate, but TA application amplified it and the neuron was more easily excitable after wash. **(D1,D2)** Yohimbine effects on the OA or TA effects were not statistically significant (Wilcoxon signed-ranks test for OA *W* = 11, *n*_s/r_ = 13, *z* = 0.37, *p* = 03557 and for TA *W* = 23, *n*_s/r_ = 9). Numbers of experiments are indicated above each bar.

**FIGURE 6:**
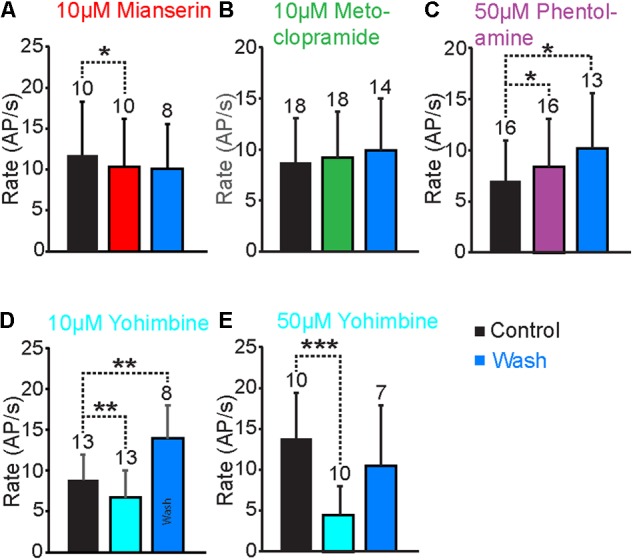
Antagonist effects on the baseline AP rate. **(A)** When the VS-3 neurons were superfused in saline with 10 μM mianserin the AP rate decreased slightly but significantly (paired *t*-test: *t* = 2.36, *df* = 9, *p* = 0.0213005). The AP rate after wash was not statistically significantly different when compared to control (*t* = 10.94, *df* = 7, *p* = 0.189257). **(B)** When metoclopramide was added to superfusion, the AP rate did not change (paired *t*-test *t* = –0.74, *df* = 17, *p* = 0.234699) and it remained unchanged after a wash (paired *t*-test *t* = –1.51, *df* = 13, *p* = 0.077481). **(C)** Superfusion in saline with phentolamine made the neurons fire slightly more (paired *t*-test *t* = –1.76, *df* = 15, *p* = 0.04939) and the AP rate increased further after wash (paired *t*-test *t* = –2.25, *df* = 12, *p* = 0.021999). **(D)** Ten micromolar yohimbine reduced the baseline spiking significantly (Wilcoxon ranked-sum test *W* = 71, *n*_s/r_ = 13, *z* = 2.46, *p* = 0.0069). After wash the baseline firing rate was significantly higher than under control conditions (Wilcoxon ranked-sum test *W* = –36, *n*_s/r_ = 8, *p* = 0.005). **(E)** Fifty micromolar yohimbine also reduced the AP rate significantly (paired *t*-test *t* = 4.45, *df* = 18, *p* = 0.000155). After wash the AP rate was not statistically different when compared to the control (paired *t*-test *t* = 0.88, *df* = 12, *p* = 0.198071). Numbers of experiments are indicated above each bar.

To identify the receptor types that responded to TA and OA in the VS-3 neurons, we tested a series of antagonists that have been widely used to investigate invertebrate and vertebrate biogenic amine receptors: mianserin, metoclopramide, phentolamine and yohimbine. We also tested chlorpromazine, but most neurons died after this antagonist was added to the superfusion and we were unable to get consistent results. Although each of these antagonists acts preferentially on a specific receptor types, none is exclusively specific (Verlinden et al., 2010; Ohta and Ozoe, 2014). We performed similar experiments as described above using pseudorandom noise stimulation. In control experiments the preparation was superfused in normal spider saline, and OA or TA was applied 60 s after the start of each experiment. Then, the antagonist was added to the superfusion and experiments with OA or TA were repeated every 15 min for up to 2 h. Finally, the preparation was washed with normal saline and responses to OA or TA were tested again every 15 min. In total, these experiments lasted for up to 5 h, and in several cases the cell died or the electrode came out of the cell before the wash was completed. Our analysis only used neurons where the baseline spike rate was close to (or above) control at the end of the experiments. Results for antagonist effects on OA and TA responses are shown in **Figure 5**, while **Figure 6** compares the baseline firing under control conditions, with the antagonist in superfusion and after wash.

Mianserin is the most efficient blocker of insect OAβ receptors (Maqueira et al., 2005; Balfanz et al., 2014) but it also inhibits other types of OA and TA receptors (Gross et al., 2015; Bauknecht and Jekely, 2017; Blenau et al., 2017a,b; Reim et al., 2017). When 10 μM mianserin was added to the spider VS-3 neuron superfusion, OA and TA induced increases in spike rate were significantly reduced in all neurons tested and recovered only slightly after wash (**Figures 5A1,A2**). When changes in spike rate induced by OA and TA application were compared in five different neurons, the mianserin effects were statistically significant. After mianserin treatment, the depolarizing synaptic potentials that occurred in response to OA and TA in control conditions, were also absent. Mianserin also slightly but significantly reduced the baseline response to the pseudorandom noise stimulation (**Figure 6A**).

Metoclopramide has been shown to act on OAβ receptors at low concentrations in some insects but not in others (Maqueira et al., 2005; Balfanz et al., 2014) and at higher concentrations in some OAα, TAR1, and TAR2 receptors (Ohta and Ozoe, 2014). In VS-3 neurons, metoclopramide was a more potent blocker of the TA than OA responses (**Figures 5B1,B2**). Although 50 μM metoclopramide reduced the OA effect on spike rate, the difference was not statistically significant. Conversely, 10 μM metoclopramide was adequate to significantly reduce the response to TA. In both cases the metoclopramide effects were at least partially reversible. Metoclopramide also inhibited the TA induced depolarizing synaptic potential (Control 4.9 ± 2.0 mV; metoclopramide 2.14 ± 1.8 mV, *p* = 0.008844, *df* = 6, *t* = 3.24, paired *t*-test), but had a smaller effect on the OA induced synaptic potential (Control 4.6 ± 2.8 mV, metoclopramide 3.4 ± 2.6 mV, *p* = 0.1341185, *t* = 1.22, *df* = 6, paired *t*-test). Metoclopramide had no effect on the baseline firing (**Figure 6B**). These findings suggest that the TA and OA responses could be at least partially mediated by two different receptor types.

Phentolamine inhibits OAβ receptors in some insects but not in others and has been shown to have agonist effects in expression systems (Maqueira et al., 2005; Chen et al., 2010; Lam et al., 2013; Balfanz et al., 2014). It is a very efficient inhibitor of honeybee TAR2 receptors and it inhibits TAR1 receptors in some insects, as well as in the tick *Rhipicephalus microplus* (Gross et al., 2015; Reim et al., 2017). Phentolamine inhibited VS-3 neuron responses to both OA and TA (**Figures 5C1,C2**). This effect was only partially reversible in most cells. The baseline spike rate was also significantly higher after phentolamine application and this effect was even larger after wash (**Figure 6C**), suggesting that phentolamine may function as an agonist in VS-3 neurons. However, OA alone produced a long lasting excitatory effect on VS-3 neurons with a significant increase in spike rate after first application that is detectable for at least 1 h (Torkkeli et al., 2011).

In vertebrates, yohimbine is an α2-adrenergic antagonist and it has a significantly higher affinity for invertebrate TAR1 and OARα than for OARβ (Maqueira et al., 2005; Lange, 2009; Balfanz et al., 2014; Reim et al., 2017). When yohimbine was added to the superfusion, VS-3 neuron response to pseudorandom noise stimulus became clearly smaller than under control conditions (**Figures 5D1,D2, 6D,E**). This effect was more pronounced with higher yohimbine concentration. However, yohimbine did not reduce the response to OA or TA (**Figures 5D1,D2**). Once yohimbine was removed from superfusion, the neurons often fired spontaneous action potentials and had a stronger response to pseudorandom stimulation than under control conditions. With 10 μM yohimbine this rebound effect after wash was statistically significant (**Figure 6D**), but removal of 50 μM yohimbine was slower, and although the rebound effect was detected in several neurons, it was not statistically significant.

## Discussion

### Phylogenetic Classification

Our phylogenetic analysis (**Figure 2**) was made using complete amino acid sequences but results are largely similar to previous reports using trimmed sequences (e.g., Dacks et al., 2013; Bauknecht and Jekely, 2017; Reim et al., 2017). As in previous studies, the basal branching of the subgroups was poorly supported by the bootstrap values, indicating that the relationships of biogenic amine receptors should be further investigated (Dacks et al., 2013; Spielman et al., 2015; Blenau et al., 2017a). However, all *C. salei* sequences were in highly supported clusters with other receptors in protostomes, most with sister groups to sequences in deuterostomes. As expected, the *C. salei* receptors were closest to other arachnid receptors, and distinct from insect receptor clades. The 5-HT_2_ and the invertebrate DAR receptor clades were found close to clades with different ligand specificity, as has been reported before (Dacks et al., 2013; Blenau et al., 2017a) suggesting that ligand specificity has varied during evolution. We did not find any *C. salei* receptors in these two clades. The sole Cs5-HT receptor was in the 5-HT_1_ clade, two dopamine receptors clearly in the DAR2 and one in the DAR1 clades.

In many earlier analyses, the TAR1 has been the closest receptors to the vertebrate α2-adrenergic receptors and the OARα closest to the α1-adrenergic receptors (e.g., Chen et al., 2010; Wu et al., 2012a). However, some more recently characterized receptors are closer to α1- or α2-adrenergic clades even though there is significant variability in their nomenclature. The *Drosophila* and *Chilo suppressalis* α2-adrenergic receptors have been named OAR3 due to their high sensitivity to OA (Wu et al., 2014; Qi et al., 2017). However, they are also very sensitive to TA, and α2-adrenergic receptors cloned from three marine invertebrates (*P. dumerilii, P. caudatus*, and *S. kowalevskii*) are more sensitive to adrenaline and noradrenaline than OA or TA (Bauknecht and Jekely, 2017). Our analysis indicates that α1- and α2-adrenergic receptors are common in arachnids, but there is no pharmacological data to support classification of these receptors and our naming is based on their position in the phylogenetic tree.

### Tissue Distribution

Most of the ten putative biogenic amine receptors were found in *C. salei* central nervous system and hypodermis transcriptomes in relatively similar amounts. However, abundances of *CsTAR1, CsDAR2A* and *B* genes were higher in the CNS than in the hypodermis while the *CsOARα* and OARβB were more abundant in the hypodermis than in the brain. Since both tissues used for RNA extraction and transcriptome creation contained muscle fibers, it is possible that some of these receptors are predominantly expressed in muscle. The tissue distributions of orthologous receptors have been studied in many insect species and they are expressed in their brain and nerve cord as well as in muscles and various internal organs (Wu et al., 2012b; Lam et al., 2013; Troppmann et al., 2014; El-Kholy et al., 2015; Blenau et al., 2017a; Kita et al., 2017; Thamm et al., 2017). Interestingly, we found only one type of tyramine receptor in the spider transcriptomes, while the nervous tissues of many other invertebrates also have TAR2 receptors (El-Kholy et al., 2015; Wu et al., 2015) and *Drosophila* has an additional TAR3 receptor type (Bayliss et al., 2013). It seems that our finding is typical for arachnids, since our GenBank search did not find any TAR2 genes from arachnids.

*In situ* hybridization experiments indicated that four biogenic amine receptors are expressed in the mechanosensory neurons of the leg hypodermis. Although many orthologous receptors in other arthropods are expressed in the antennae or other organs that contain mechano- and chemosensory neurons, to our knowledge there is only one earlier report that has specifically detected one receptor type (TAR1) in the chemosensory neurons of the moth, *Mamestra brassicae*, antennae (Brigaud et al., 2009). However, there is ample evidence that biogenic amines, especially OA and TA, modulate arthropod mechano- and chemosensory neurons (Grosmaitre et al., 2001; Roeder, 2005; Widmer et al., 2005; Zhukovskaya and Polyanovsky, 2017). Our findings here begin to explain why the same ligand can induce different effects in the same neuron.

Previously, an antibody against the third intracellular loop of the *Drosophila* OARα (Han et al., 1998) was tested in immunohistochemistry on the *C. salei* patellar hypodermis and in Western blot against the brain homogenate (Widmer et al., 2005). It produced a band at about 75 kDa in Western blot and specific immunolabeling on the axonal region of the mechanosensory neurons. Several *C. salei* biogenic amine receptor sequences match partially to the *Drosophila* residues that were used to create this antibody. The closest match was for CsOARα, but since this receptor was not expressed in the sensory neurons, the *Drosophila* antibody probably labeled one or more of the receptor types that were found by *in situ* hybridization.

### Modulation of VS-3 Neurons by Biogenic Amines

Our electrophysiological experiments revealed two distinctly different responses to adrenergic antagonists: (1) Mianserin, phentolamine, and metoclopramide reduced the change in sensitivity normally caused by OA or TA. Yohimbine had no effect on this response. (2) Yohimbine and, to a lesser extent, mianserin reduced the baseline sensitivity and removal of yohimbine often caused a rebound increase in sensitivity. This clearly indicates that VS-3 neurons have a constitutively active receptor type. There is very little direct evidence on the second messenger mechanisms or pharmacology of any arachnid biogenic amine receptors. Hence, we will discuss our findings based mostly on information from other invertebrate receptors.

CsOARβ shares high similarity with several insect β-adrenergic-like receptors that are highly sensitive to OA and less sensitive to TA. They are coupled to G_s_ proteins and, in expression systems, stimulate cAMP production (Balfanz et al., 2005, 2014; Maqueira et al., 2005; Chen et al., 2010; Wu et al., 2012b). The most potent inhibitor of the insect OARβ is mianserin while their sensitivity to phentolamine and metoclopramide is lower and yohimbine has no effect (Maqueira et al., 2005; Wu et al., 2012b; Balfanz et al., 2014). It is likely that the VS-3 neuron response to OA and TA is mostly mediated by the CsOARβB. This is also supported by previous findings on spider mechanosensory neurons where the cAMP analog 8-Br-cAMP produced a comparable increase in sensitivity to OA (Widmer et al., 2005; Torkkeli et al., 2011). However, Rp-cAMPS, which acts as a competitive antagonist of cAMP-induced activation of PKA, did not significantly reduce the OA response and this response was inhibited by CaMKII inhibitor KN-62, suggesting that the OA effect is mediated by Ca^2+^ (Torkkeli et al., 2011). Nevertheless, PKA activation is only one of several mechanisms mediated by cAMP; it can activate exchange (Epac) proteins or open Ca^2+^ and other ion channels and β-adrenergic receptors also activate CaMKII (Lim et al., 2014).

The VS-3 neuron responses to OA and TA are probably mediated by the same receptors, since most invertebrate OA and TA receptors can be activated by both ligands, the responses recorded here were very similar and they were reduced by the same antagonists. The stronger effect of metoclopramide on TA than OA response may be caused the cell’s more robust response to OA that is more difficult to block. It is also possible that TA activates another pathway via CsTAR1 or Csα2-adrenergic receptors. However, these receptor types are generally inhibited by yohimbine, which did not block the OA or TA responses in VS-3 neurons.

It is not clear if VS-3 neuron responses to DA or 5-HT are also mediated by CsOARβ. DA has been tested on a small number of OAβ receptors and they are significantly less sensitive to DA than OA or TA (Chen et al., 2010; Wu et al., 2012b). We did not find DA receptors in VS-3 neurons by *in situ* hybridization, so it is very likely that DA activates the OAβ receptors. 5-HT has only been tested in a small number of OA or TA receptors and found ineffective (e.g., Han et al., 1998; Poels et al., 2001; Balfanz et al., 2005; Wu et al., 2012a,b). Although we found that the *Cs5-HTR_*1*_* is expressed in VS-3 neurons it is unlikely to mediate the excitatory 5-HT response, since these receptor types usually mediate inhibitory responses. So, these neurons may have an additional 5-HT receptor type that has not yet been identified.

The effect on baseline sensitivity is likely to be mediated by the CsTAR1 and/or Csα2-adrenergic receptors. These receptor types are coupled to G_i_ and/or G_q_ proteins and thus inhibit cAMP synthesis and/or mobilize intracellular Ca^2+^ (Robb et al., 1994; Poels et al., 2001; Mustard et al., 2005; Wu et al., 2012a, 2014; Ohta and Ozoe, 2014; Gross et al., 2015; Qi et al., 2017). Both pathways are inhibited by yohimbine and less efficiently by mianserin (Robb et al., 1994; Poels et al., 2001; Gross et al., 2015; Bauknecht and Jekely, 2017; Reim et al., 2017). Since the reduced baseline firing can only be caused by inhibition of an excitatory effect, it is likely that this baseline activity is maintained by ligand binding to CsTAR1 or Csα2-adrenergic receptor that activates G_q_ proteins.

The 5-HT_1_ receptors that have been tested on expression systems are purely inhibitory and coupled to G_i_ or G_i/o_ proteins; the former inhibiting cAMP and the latter closing Ca^2+^ channels (Saudou et al., 1992; Chen et al., 2004; Thamm et al., 2010; Troppmann et al., 2010). Therefore, our *in situ* hybridization results indicate that the spider mechanosensory neurons have three types of inhibitory biogenic amine receptors. Although none of the agonists that we tested produced inhibitory responses, we show here, and have reported before, that after a saturating concentration is reached, application of higher concentrations of OA or TA produces a smaller effect (Torkkeli et al., 2011). Reduced effects by high biogenic amine concentrations have also been reported in several other preparations before (e.g., Reyes-Colon et al., 2010; Jezzini et al., 2014). It is possible that the receptors desensitize when agonist concentration is high, but another explanation is that the G_i_ proteins are activated by high concentration and reduce [cAMP]. However, a similar theory was tested in functionally expressed *Bombyx mori* OAβ receptors, where a comparable concentration-dependent effect was seen (Chen et al., 2010). Blocking of G_i_ protein with pertussis toxin (PTX) elevated cAMP production at all concentrations, but the response was still smaller at high concentrations, suggesting that endogenous G_i_ proteins were inhibited. Whether the inhibitory G proteins are activated at all ligand concentrations or primarily at high concentration, their major function appears to be to regulate the stimulatory effect of OA or TA mediated by excitatory OAβ receptors.

The physiological significance of the biogenic amine receptors depends on the availability of various amines. Several 5-HT and OA immunoreactive somata and processes were found in the *C. salei* subesophageal ganglia but not initially in any of the peripheral nerves (Seyfarth et al., 1993; Fabian and Seyfarth, 1997; Barth, 2002). However, OA was later found in some of the fine efferent fibers that project to the VS-3 and other mechanosensory neurons of the leg (Widmer et al., 2005). Since TA is the precursor of OA, it must also be present in the *C. salei* CNS and maybe also in the periphery. To our knowledge, dopamine distribution in the spider CNS has not been investigated. All biogenic amines act as neurohormones released to the circulation as well as neurotransmitters or modulators that act locally by release from nerve endings (Roeder, 2005). It is possible that OA released form the efferent nerve endings acts on the OAβ receptors causing a rapid effect while circulating OA maintains the baseline sensitivity.

## Conclusion

In conclusion, we have established that arachnids have similar groups of biogenic amine receptors to other protostome invertebrates, but they lack two clades. We also clarify that, contrary to previous assumptions, arachnids and many other invertebrates have both α1- and α2-adrenergic receptors. Using *in situ* hybridization, we found four different biogenic amine receptors expressed in spider mechanosensory neurons, and with electrophysiological and pharmacological tools we discovered possible roles of these receptors in modulating neuronal sensitivity. These neurons have a constitutively active receptor type that adjusts the baseline sensitivity to a level appropriate for the behavioral state of the animal while another type of receptor regulates rapid and large changes in sensitivity. Together, these mechanisms probably allow the spider to monitor its environment and fine-tune its sensory neurons to resting or alert states, while still able to detect sudden changes in its surroundings.

## Author Contributions

VS and SM performed the electrophysiological experiments. HL designed the primers and did the *in situ* hybridization and RACE. AF identified the sequences from the transcriptome libraries and wrote all custom written software. PT designed the study, performed the phylogenetic analysis, and wrote the manuscript. All authors participated in analysis and interpretation of the data and read and approved the final manuscript.

## Conflict of Interest Statement

The authors declare that the research was conducted in the absence of any commercial or financial relationships that could be construed as a potential conflict of interest.

## Footnotes

1 http://mafft.cbrc.jp/alignment/server/

2 http://vassarstats.net/
