# Supplementary material for: Multiple Biogenic Amine Receptor Types Modulate Spider, Cupiennius salei, Mechanosensory Neurons
Source: Front Physiol. 2018 Jul 9;9:857. doi: 10.3389/fphys.2018.00857 (PMC6052906; doi:10.3389/fphys.2018.00857)
Supplement: Supplementary file 1 [file Table_1.PDF]

Additional file 1: List of genes used for phylogenetic analysis

Phylum/class	Species	Protein	Uniprot / *NCBI code*
**Arthropoda/Insecta**	*Anopheles gambiae*	OARα (AgOAR45A)	Q7PRZ8
	Apis mellifera	OARα (AmOa1)	Q70TB0
		OARβ1	W0U8S1
		α2-Adrenergic	A0A088AGH3
		TAR1 (AmTyr)	Q9NG02
		TAR2	A1X3A8
		5-HTR1	D2U6U8
		5-HTR2α	E5BBP1
		5-HTR7	Q0P7L6
		DAR1	O44198
		DAR2	Q8T0Y4
		DAR3	Q56JK9
	Bactrocera dorsalis	OARβ1 BdOctbR1	XP_011212557
	Bombyx mori	OARα	Q08JR9
		OARβ (BmOAR2)	D5MQ02
		TAR1 (B96Bom)	Q75Q62
		TAR2 (BmTAR2)	D1MW42
		5-HTR1A	A0A0A7LX89
		DAR1	A9ZLX6
		DAR2	A9ZLX7
	Calliphora vicina	5-HTR2α	I7JA47
		5-HTR7	I7IA71
	Chilo suppressalis	OARα (CsOA1)	G5DE89
		OARβ2 (CsOA2B2)	G3M4F8
		TAR1	H9TVJ1
		TAR2	E7CHA2
		OARα2-3S	A0A068AZY5
	Drosophila melanogaster	OARα (OAMB)	Q9VDJ6
		OARβ1	Q9VCZ3

**Phylum/class**	**Species**	**Protein**	**Uniprot / *NCBI code***
		TAR1 (Oct-TyrR)	P22270
		α2-Adrenergic	Q9VE32
		TAR2	Q9VEG1
		TAR3	Q9VEG2
		5-HTR1B	P28286
		5-HTR2A	Q24511
		5-HTR7	P20905
		DAR1-1 (DmDOP1R1)	P41596
		DAR1-R1-2 (DmDOP1R2)	Q24563
		DAR2 DmDop2R	Q8IS44
	Musca domestica	OARα	A0A1I8MWJ9
		α2B-Adrenergic	A0A1I8N9Y1
	Locusta migratoria	TAR1	Q25321
	Periplaneta americana	OARα (PaOa1)	Q7YWB7
		TAR1 (PeaTyr)	B2G4W4
		DAR2 (PeaDOP2)	V6DNI7
		5-HTR1	C1L3G5
	Schistocerca gregaria	OARα	D5JAN7
		OARβ	D5JAN8
**Arthropoda/myriapoda/chilopoda**	*Strigamia maritima*	GPCR	T1IJG9
**Arthropoda/Chelicerata/Merostomata**	*Limulus polyphemus*	α-2C adrenergic<br>5-HT	*XP_013776216*<br>*XP_013789239*
**Arthropoda/Chelicerata/Arachnida**	*Amblyomma cajennense*	DAR1β	A0A023FH63
	Ixodes ricinus	DAR2	A0A147BLM1
	Ixodes scapularis	OARα (OAMB)	B7PJX6
		GPCR	B7PKJ3
		OARβ	B7Q337
		TAR1	B7QL84
		5-HTR	B7Q8G2

**Phylum/class**	**Species**	**Protein**	**Uniprot / *NCBI code***
		DAR1β	B7P6V6
	Metaseiulus (Galendromus) occidentalis	OAR1	*XP_003741991.1*
		OARβ2	*XP_018493636.1*
		α1A-Adrenergic	*XP_003748403.1*
	Parasteatoda tepidariorum	GPCR#9	*XP_015928138.1*
		α1A-Adrenergic	*XP_015904375.1*
		α2Db-Adrenergic	*XP_015914395.1*
		DAR1	*XP_015927542.1*
		DAR2A	*XP_015930262.1*
		DAR2B	*XP_015920481.1*
		5-HTR2B	*XP_015911286.1*
		OARβ2	*XP_015922383.1*
		OARβ3	*XP_015929036.1*
		GPCR#18	*XP_015917055.1*
	Rhipicephalus appendiculatus	α2B-Adrenergic	A0A131Z032
	Rhipicephalus (boophilus) microplus	OARα2	H9B3X9
		5-HTR	Q6VWJ2
		GPCR	O77254
		DAR1	H9B3Y5
		DAR2	H9B3Y3
		TAR1	A7TZ10
	Stegodyphus mimosarum	α1A-Adrenergic	A0A087UJX9
		DAR1	A0A087TFH8
		OARβ2A	A0A087T4K2
		OAR2 (α2-Adrenergic)	A0A087TC89
		TAR2	A0A087T9F7
		5-HTR2A	A0A087TLT9
		5-HT2B	XP_015911286
		OARβ2B	A0A087T4X6
	Tetranychus urticae	GPCR1	T1KND7
		GPCR2	T1JR78
		α2C-Adrenergic	*XP_013776216.1*
		5-HTR	*XP_013789239.1*
**Arthropoda/Crustacea**	*Daphnia magna*	α1B-Adrenergic	A0A0P5NUL6

**Phylum/class**	**Species**	**Protein**	**Uniprot / *NCBI code***
		α2C-Adrenergic	A0A0P5VX37
		DAR2	A0A0P6DQ39
	Panulirus interruptus	DAR1α ($D1_{αpan}$)	Q2V625
		DAR1β ($D1_{βpan}$)	Q2V624
		DAR2 ($D2_{αpan}$)	Q09HM5
		5-HTR1α	Q6QR16
		5-HTR2β	Q6QA79
	Procambarus clarkii	5-HTR1α	B0F4P9
		5-HTR2β	B0F4P8
**Annelida/Polychaeta/Nereididae**	*Platynereis dumerilii*	α1-Adrenergic (OARα1)	A0A1J0F5Z2
		α2-Adrenergic	A0A1J0F5X6
		TAR1	A0A0K0PUV5
		TAR2	A0A1J0F4P1
**Priapulida**	*Priapulus caudatus*	α1A-Adrenergic	*XP_014662992*
		α2C-Adrenergic	*XP_014681069*
**Mollusca/gastropoda**	*Aplysia californica*	OARα (Apoa)	Q9NHF3
		5-HTRA1	O76267
		5-HTRB1	Q16950
		5-HTRB2	Q16951
	Aplysia kurodai	5-HTR1 5-HTAp1AC1	C7SLZ0
	Lottia gigantean	GPCR	V4A2Q6
	Lymnae stagnalis	OARα LymOA1	O77408
		5-HTR1	Q25414
		5-HTR2	O61232
**Hemichordata/Enteropneusta**	*Saccoglossus kowalevskii*	α1-Adrenergic	A0A0U2M149
		OARα	*XP_006823182*
		OARβ	*XP_002733926*
		α2C-Adrenergic	*XP_002734932*

Phylum/class	Species	Protein	Uniprot / *NCBI code*
		TAR1	*XP_002742354*
		TAR2A	*XP_002734062*
		TAR2B	*XP_006812999*
**Chordata/mammalia**	*Homo sapiens*	α1A-Adrenergic	P35348
		α1B-Adrenergic	P35368
		α1D-Adrenergic	P25100
		α2A-Adrenergic	P08913
		α2C-Adrenergic	P18825
		β1-Adrenergic	P08588
		β2-Adrenergic	P07550
		β3-Adrenergic	P13945
		5-HTR1B	P28222
		5-HT2A	P28223
		5-HTR4	Q13639
		5-HTR5A	P47898
		5-HTR6	P50406
		5-HTR7	P34969
		DAR2	P14416
		DAR3	P35462
		DAR4	P21917

OAR = Octopamine receptor; TAR = tyramine receptor; DAR = dopamine receptor; 5-HTR = serotonin receptor; NCBI codes are shown in italics for sequences where Uniprot codes are not available.
